# The response of diabetic foot to a new type of dressing

**DOI:** 10.1186/1755-7682-5-33

**Published:** 2012-12-18

**Authors:** Katarzyna Skórkowska-Telichowska, Anna Kulma, Jan Szopa

**Affiliations:** 1 Department of Endocrinology, Clinic of Internal Medicine, IVth Clinical Military Hospital, R. Weigla 5 Street, Wrocław, 50-981, Poland; 2 Faculty of Biotechnology University of Wroclaw, Przybyszewskiego 63, Wroclaw, 51-147, Poland

**Keywords:** Leg ulcer, Diabetes, Linen dressing, Therapy, Genetic engineering

## Abstract

**Background:**

FlaxAid is a newly developed type of dressing enriched in particular flavonoids through genetic engineering of flax plants that exhibit health-promoting activities due to their strong antioxidant properties. The purpose of the current study was to assess the clinical efficacy of the FlaxAid bandage therapy for a patient affected with a diabetic foot ulcer which was unresponsive to previous treatments. The patient was treated with FlaxAid bandages for 12 weeks and the size and properties of the wound were routinely observed and recorded. Due to the the clinical picture of the wound study design was adopted whereby the comparative treatment was cotton gauze wetted with isotonic salt solution.

**Findings:**

Following therapy, the foot ulcer decreased in size, despite the decompensation of advanced diabetes. It is believed that the beneficial nature of FlaxAid is derived from its high level and broad spectrum of antioxidants.

**Conclusions:**

The FlaxAid dressing provides a novel and effective method for the treatment of diabetic foot ulcers. This study presents a preliminary pilot investigation and a larger number of subjects need to be included within the study in order to draw firm clinical conclusions. Efforts to this effect are currently under way.

## Findings

### Introduction

The risk of developing foot ulcers in people affected with diabetes is 12-25%, and such patients are 30–40 times more likely to undergo leg amputation than people with similar ulcers but without diabetes. In developed countries, diabetic foot syndrome has become the most frequent cause of non-traumatic loss of legs [1-6].

We assessed a new material and method for the treatment of foot ulcers based on products from transgenic flax plants that overproduce various antioxidative compounds such as phenolics in the fibres, unsaturated fatty acids in the seeds, and the antioxidant lignans in the seedcakes. Coordinative use of the fibres, oil emulsion and seedcake extract from these plants was expected to promote the healing of chronic skin ulcerations. The purpose of the current study was to assess the healing properties of products derived from transgenic flax plants towards chronic skin ulcers in diabetic affected patients.

### Materials and methods

#### Subject

The subject who participated in the study was a 56-year old patient with diabetes (initials S.P., male, diagnosis 16 years prior), who visited the endocrinology out-patient clinic to receive treatment for a recurrent non-healing ulcer on his right foot in the plantar region. The condition had lasted for three years at that point.

#### Signs and symptoms

The anamnesis and physical examination revealed diabetes-related complications, including advanced, proliferative retinopathy and maculopathy in both eyes, diabetic nephropathy (patient requiring haemodialysis), diabetic polyneuropathy of the central and peripheral nervous systems (patient needs to use a wheelchair), macroangiopathy (coronary artery disease, post-TIA state) and diabetic foot syndrome.

The ulcer was oval in shape with sharp protruding edges, and was deep and clean, without the presence of necrotic tissue, fibrin masses or macroscopic features of infection. Over the previous 3 years, the ulcer had become deeper and wider, despite continuous local and general therapy. The patient has used almost all dressings available in our country and a majority of them have caused a local allergic reaction of the skin adhering the ulceration. The one dressing the patient tolerated best was gauze wetted with 0,9% NaCl. The patient reported no accompanying pain due to diabetic and uraemic polyneuropathy. The course of the diabetes was labile, and despite intensive insulin therapy, the control of systemic glucose concentration was unsatisfactory. The wound pathophysiology was diagnosed using the patients’ medical history, physical examination, Doppler sonography of the leg arteries, and segmentary measurement of systolic pressure on lower extremities with ankle-brachial index (ABI) measurement.

The routine laboratory tests revealed diabetic decompensation and related complications with results as follows: HbA1c: 9.1% (N<6.5%), fasting glucose: 197 mg% (N:60–99 mg%), creatinine: 8.4 mg% (N:0.81- 1.44mg%), uric acid: 124 mg% (N:10–50 mg%), total protein: 6.5 g/l (N: 6.4-8.3 g/dl), total cholesterol: 231.5 mg% (N: 125–200 mg%), LDL-cholesterol: 170.8 mg% (N:0–130 mg%), HDL-cholesterol: 21.9 mg% (N: 40-80mg%), triglicerydes: 200mg% (N:40-140mg%), fibrinogen: 6.1g/l (N<4.5 g/l), CRP: 164 mg/l (N:<30 mg/l), erythrocytes: 3.10 T/l (N: 4.2-6.0), hemoglobin: 8.3 (N: 14–18 g/%), hematocrit: 26.8%(N: 40-54%), Fe: 24 ug/dl (N:53–167 ug/dl), platelets: 625 g/L (N: 14–440 g/L). Blood culture and ulcer culture were each performed three times and the results were negative. ABI (right) was 0.6, ABI (left) was 0.8 (N>1.0).

The physical examination, duplex ultrasonography of the leg’s vessels and the segmentary measurement of systolic pressure on lower extremities revealed problems with small vessels (below the knee) as a cause of ulceration. Invasive diagnostic procedure (arteriography) wasn’t performed because of the general state of the patient’s health and lack of patient’s permission for arterial revascularisation. The above mentioned lab results together with classic diagnostic tests indicated that diabetic foot syndrome was the primary pathology.

#### Preparation of the FlaxAid dressing

The FlaxAid fabric was prepared from raw yarn using the standard weaving method. The linen dressing was sterilized by autoclaving at 120°C for 20min. Where indicated, the linen dressing for the wound treatment was covered with 2ml of sterile seedcake extract or with 2ml of oil emulsion.

A flax oil emulsion was prepared by mixing soybean lecithin with flax oil. An aqueous phase containing glycerol was then added to the resulting mixture. The mixture was then sonicated in a Microson ultrasonic cell disruptor for 10min at 4W and then filtered through sterile Acrodisc 0.22 mm filters. The final concentrations of the chemicals in the resultant emulsion were: 1% lecithin, 2.5% flax oil, 2.5% Tween 80, and 2.5% glycerol.

The seedcake extract was prepared by extracting defatted seedcakes thrice with 80% methanol (v/v) for 15min at 70°C. After methanol evaporation the extract was hydrolysed in 0.3M NaOH for 2 days at room temperature followed by neutralization using 2M hydrochloric acid. Following centrifugation, the supernatant was filtered through an Acrodisc 0.22mm filter and sterilized by autoclaving at 120°C for 20min. The concentration of the components was determined by HPLC analysis using internal standards. Prior to clinical use, the extract was diluted to a final concentration of 5 mg/ml of SDG.

#### Chemical constituents of the FlaxAid dressing

The FlaxAid dressing contains: cellulose (680 mg/g); pectins (35 mg/g); lignans (25 mg/g); soluble (0.05 mg/g) and ester-bound phenolics (1.3 mg/g); and carotenoid-lutein (0.29 μg/g). With regards to the phenolics, the most aboundant in flax fibers are vanilin (0.48mg/g) and ferulic (0.35 mg/g) acid. Other phenolics such as coumaric, vanilic and 4-hydrohybenzoic acids and syringialdehyde are present. All of the phenolics have been previously proved to exhibit strong antioxidative properties and positively influence cell proliferation in vitro.

The oil emulsion contains flax oil and provides unsaturated fatty acids within the FlaxAid fibre dressing. The main constituents of the oil emulsion include linoleic acid (645 mg/g), oleic acid (194 mg/g), and linolic acid (22mg/g). Flax oil also contains relatively large amounts of antioxidants. These include hydrophobic antioxidants such as γ-tocopherol (0.6mg/g), plastochromanol (0.5 mg/g) and lutein (0.04 mg/g) as well as hydrophilic phenolic acids (6mg/g) consisting mainly of ferulic, coumaric, chlorogenic and caffeic acids as well as vanillin.

The seedcake extract provides mainly antioxidants which can also affect cell proliferation. The main constituents of seedcakes are: lignans such as secoisolariciresinol diglucoside (SDG) (58 mg/g); and phenolic acids such as ferulic acid (0.32 mg/g), ferulic acid glucoside (5.48 mg/g), coumaric acid (0.03 mg/g), coumaric acid glucoside (0.71 mg/g), caffeic acid (0.032 mg/g) and caffeic acid glucoside (0.547 mg/g).

#### Application

The standard FlaxAid bandage therapy consists of three sequentially applied stages: the linen layer stage, the linen plus oil emulsion stage, and the linen plus seedcake extract stage. For this study, the treatment was divided into three stages (labeled first, second and third), each lasting four weeks. Stage zero was cotton gauze wetted with isotonic salt solution, which had been locally applied to the ulcer for a period over 12 weeks during the 3 year period prior to the FlaxAid application and had in essence served as a reference for the study. The patient did not have another skin ulcer thus making it impossible to use direct comparison with a negative and positive control approach.

After each week, during a consultation, a physician performed an evaluation of the ulcer, measured the ulcer using sterile dressings with a demarcated millimeter scale and prepared photographic and descriptive documentation. The patient filled in a questionnaire the day prior to each visit and this was also taken into consideration for the clinical evaluations.

The dressings were changed every 24 hours, and the first application in each stage was done by qualified hospital personnel. The patient himself changed the dressings thereafter, having been thoroughly instructed by a qualified nurse during the weekly visits.

The study was approved by the local bioethics committee. The patient was provided with written information on the purpose and design of the study.

#### Clinical evaluation

To evaluate the clinical changes associated with the FlaxAid therapy, several parameters were considered: the change in wound exudates, the changes in the fibrin and granulation levels within the ulcer, and the size of the ulcer.

### Results

Following the zero stage, slight but negative changes were observed in all the considered parameters, suggesting that the common treatment (cotton gauze wetted with isotonic salt solution) had no positive impact on ulcer healing within the time-frame of the study.

After the first stage (FlaxAid with isotonic salt solution), the ulcer became shallower with a slight reduction in the wound size. In addition, a minimal decrease in the level of exudates was also observed.

After the second stage (FlaxAid with oil emulsion), the ulcer was still clean, there was less fibrin, and there was new red granulation tissue in the wound bed. The wound had become drier with a significant reduction in wound size due to marginal epithelium growth.

**Figure 1:**
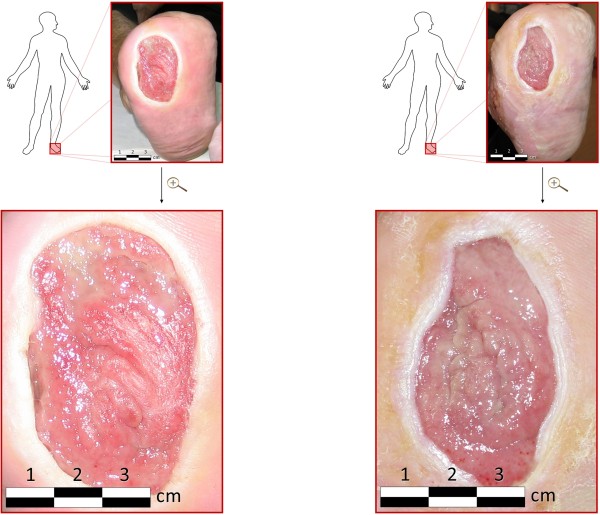
**The patient’s diabetic foot ulcer before the start of the study (a) and after the 12-week treatment(b) with newly developed type of dressing enriched in health-promoting flavonoids through genetic engineering of flax plants.** After 12 weeks lasting therapy, the foot ulcer decreased in size, despite the decompensation of advanced diabetes.

All these effects were also visible in the third stage (FlaxAid with seedcake extract). In addition, the wound size diminished due to reepithelialisation (see Figure 1). The size of the wound during the zero stage of treatment was ~19.60 cm^2^ with the size not changing significantly over the 12-week period prior to FlaxAid therapy. During application of the FlaxAid dressing over the concomitant 12-week period, the size of the ulcer wound was as follows: after the first stage ~18.00 cm^2^, after the second stage ~16.45 cm^2^, and after the third stage ~12.25 cm^2^. These changes were determined to be statistically significant compared to the initial wound size measurements prior to and after the zero stage of treatment. In addition, significant changes in wound parameters were discerned between the different stages of FlaxAid dressing applications. The laboratory parameters were stable during the experiment.

### Discussion

It has previously been reported that phenylpropanoid compounds, in particular flavonoids, exhibit health-protecting activity due to their strong antioxidant properties, which might have great significance for the treatment of chronic ulceration [7-9]. Following 12 weeks of local treatment with the FlaxAid bandage the treated ulcer became smaller in size and remained clean. This is especially significant given the issues with concomitant diabetes decompensation: numerous complications that make wound healing more difficult. It is believed that the beneficial nature of FlaxAid is derived from its high levels of broad spectrum antioxidants. The patient is still receiving local FlaxAid therapy. Although the current study represents a single patient treated with the FlaxAid dressing, preliminary results appear to be positive with a larger treatment group. Efforts are currently underway to enroll more subjects in order to form firm conclusions and those results will be published in the near future. In addition, the long-term outcome of the product is currently being investigated. Finally, a series of studies are currently underway in order to determine the clinical efficacy of the FlaxAid dressing in treating different skin wound conditions.

## Ethics approval

Ethics approval was obtained from the local bioethics committee (Bioethics Committee, Łódź, no RNN/441/08/KB). The patient was provided with written information on the purpose and design of the study and an informed consent was acquired.

## Competing interests

None of the authors declared any conflict of interests.

## Authors’ contributions

KST Conception and design, acquisition of data, clinical evaluation, analysis and interpretation of data, drafting the article. AK Preparation of flax dressings, analysis and interpretation of data. JS Conception and design, preparation of flax dressings, critical revision of the article, final approval of the version to be published. All authors read and approved the final manuscript.

## Source of funding

The research on the composition of flax products was done in a course of grants NR 12-0009-06, NR 12017110 from the Ministry of Education and Sciences and UDA-POIG.01.04.00-22-022/11-00 from the EU Innovative Economy Programme.
